# Kinetic regulation of MXene with water-in-LiCl electrolyte for high-voltage micro-supercapacitors

**DOI:** 10.1093/nsr/nwac024

**Published:** 2022-02-23

**Authors:** Yuanyuan Zhu, Shuanghao Zheng, Pengfei Lu, Jiaxin Ma, Pratteek Das, Feng Su, Hui-Ming Cheng, Zhong-Shuai Wu

**Affiliations:** State Key Laboratory of Catalysis, Dalian Institute of Chemical Physics, Chinese Academy of Sciences, Dalian 116023, China; State Key Laboratory of Catalysis, Dalian Institute of Chemical Physics, Chinese Academy of Sciences, Dalian 116023, China; Dalian National Laboratory for Clean Energy, Chinese Academy of Sciences, Dalian 116023, China; State Key Laboratory of Catalysis, Dalian Institute of Chemical Physics, Chinese Academy of Sciences, Dalian 116023, China; State Key Laboratory of Catalysis, Dalian Institute of Chemical Physics, Chinese Academy of Sciences, Dalian 116023, China; University of Chinese Academy of Sciences, Beijing 100049, China; State Key Laboratory of Catalysis, Dalian Institute of Chemical Physics, Chinese Academy of Sciences, Dalian 116023, China; University of Chinese Academy of Sciences, Beijing 100049, China; State Key Laboratory of Catalysis, Dalian Institute of Chemical Physics, Chinese Academy of Sciences, Dalian 116023, China; University of Chinese Academy of Sciences, Beijing 100049, China; Shenyang National Laboratory for Materials Science, Institute of Metal Research, Chinese Academy of Sciences, Shenyang 110016, China; Faculty of Materials Science and Engineering/Institute of Technology for Carbon Neutrality, Shenzhen Institute of Advanced Technology, Chinese Academy of Sciences, Shenzhen 518055, China; Shenzhen International Graduate School, Tsinghua University, Shenzhen 518055, China; State Key Laboratory of Catalysis, Dalian Institute of Chemical Physics, Chinese Academy of Sciences, Dalian 116023, China; Dalian National Laboratory for Clean Energy, Chinese Academy of Sciences, Dalian 116023, China

**Keywords:** MXene, micro-supercapacitors, water-in-LiCl, aqueous high voltage, wide temperature

## Abstract

MXenes are one of the key materials for micro-supercapacitors (MSCs), integrating miniaturized energy-storage components with microelectronics. However, the energy densities of MSCs are greatly hampered by MXenes’ narrow working potential window (typically ≤0.6 V) in aqueous electrolytes. Here, we report the fabrication of high-voltage MXene-MSCs through the efficient regulation of reaction kinetics in 2D Ti_3_C_2_T*_x_* MXene microelectrodes using a water-in-LiCl (WIL, 20 m LiCl) salt gel electrolyte. Importantly, the intrinsic energy-storage mechanism of MXene microelectrodes in WIL, which is totally different from traditional electrolytes (1 m LiCl), was revealed through *insitu* and *exsitu* characterizations. We validated that the suppression of MXene oxidation at high anodic potential occurred due to the high content of WIL regulating anion intercalation in MXene electrodes, which effectively broadened the voltage window of MXene-MSCs. Remarkably, the symmetric planar MXene-MSCs presented a record operating voltage of 1.6 V, resulting in an exceptionally high volumetric energy density of 31.7 mWh cm^−3^. With the ultra-high ionic conductivity (69.5 mS cm^−1^) and ultralow freezing point (−57°C) of the WIL gel electrolyte, our MSCs could be operated in a wide temperature range of −40 to 60°C, and worked for a long duration even at −40°C, demonstrative of its practicality in extreme environments.

## Teaser

High-voltage aqueous MXene symmetric planar micro-supercapacitors based on water-in-LiCl electrolyte were developed, which presented a record operating voltage of 1.6 V and a wide temperature range of −40°C to 60°C.

## INTRODUCTION

With the rapid miniaturization of electronics, such as implantable medical chips, portable/wearable devices and the Internet of Things, the demand for microscale electrochemical energy-storage devices, including micro-batteries (MBs) and micro-supercapacitors (MSCs) with high energy density, flexibility, reliable safety features and low environmental impact has increased [1–3]. Compared to MBs, MSCs have considerable merits of high rate capability and power density in addition to an ultra long lifespan [2,4]. Furthermore, in contrast to organic MSCs, aqueous MSCs are non-flammable, environmentally friendly and safe for handling [5,6], making them better choices for practical applications.

MXenes, a family of 2D transition metal carbides and nitrides with >30 species [7], are emerging as high-performance electrode materials. Among MXenes, the most commonly synthesized and well-studied member Ti_3_C_2_T*_x_* possesses high electronic conductivity (∼10 000 S cm^−1^) and charge-storage capacity (∼1500 F cm^−3^) thanks to the presence of abundant electrochemically active sites and intercalation of ions between its layers [8,9]. However, an MXene electrode is easily oxidized at high anodic potential in aqueous electrolytes and its operating voltage is normally limited by the electrochemical thermodynamic stability window of water (1.23 V) [10,11], thus resulting in small operating voltages (typically ≤0.6 V) [12–15]. In addition, aqueous electrolytes freeze easily at sub-zero temperatures, leading to a sharp decline in ionic conductivity and flexibility [16,17]. Conversely, at high temperatures, the structure of electrolytes becomes so unstable that it is difficult to retain internal water molecules because of volatility. Recently, some concentrated aqueous ‘water-in-salt’ (WIS) electrolytes have been shown to broaden the electrochemical window of aqueous electrolytes (e.g. >3.0 V) [18–20]. Nevertheless, MXene-based MSCs with a wide temperature range and high voltage have not yet been achieved using aqueous electrolytes. Besides, the fundamental research on the charge-storage mechanism of MXene in concentrated aqueous electrolytes is sparse.

Herein, we report the development of a low-cost and environmentally friendly water-in-LiCl (WIL) salt electrolyte to regulate the reaction kinetics of MXene electrodes and electrolytes, which not only broadens the operation voltage of MXene-based MSCs (MXene-MSCs) by inhibiting oxidation at high potential, but also allows operation in a wide range of temperatures owing to a low freezing point. The as-fabricated symmetric planar aqueous MXene-MSCs with WIL electrolyte achieved an operating voltage of 1.6 V, high volumetric capacitance of 89.2 F cm^−3^ and energy density of 31.7 mWh cm^−3^ at room temperature. The low freezing point (−57°C) of the WIL gel electrolyte also enabled MXene-MSCs to operate stably in a wide temperature range going as low as −40°C. The scalability and flexibility of MXene-MSCs make them ideal for integration with wearable microelectronics.

## RESULTS AND DISCUSSION

### Physicochemical properties of the WIL electrolyte

**Figure 1.**
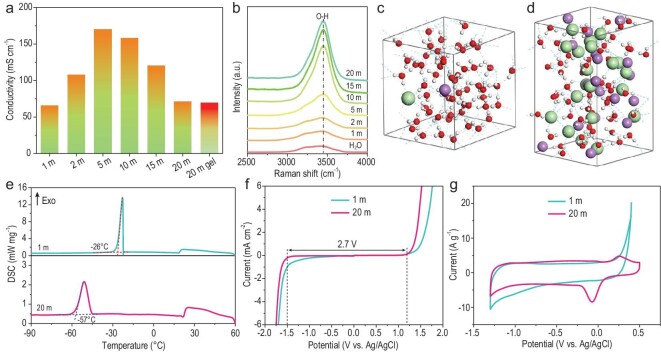
Characteristics of WIL electrolytes. (a) Ionic conductivities of 1, 2, 5, 10, 15 and 20 m LiCl electrolytes and 20 m LiCl-gel electrolyte. (b) Raman spectra of 1, 2, 5, 10, 15 and 20 m LiCl electrolytes compared with pure H_2_O. Snapshots of equilibrium trajectories of (c) 1 m and (d) 20 m LiCl electrolytes. Atom colors: Li, purple; Cl, green; O, red; H, white. (e) Differential scanning calorimetry (DSC) curves of 1 and 20 m LiCl-gel electrolytes. (f) ESW of 1 and 20 m LiCl electrolytes. (g) Cyclic voltammetry (CV) curves of MXene electrode measured in 1 and 20 m LiCl electrolytes.

Taking into consideration the economic cost and solubility (Supplementary Tables S1 and S2), we chose LiCl salt for preparing WIS electrolytes and 20 m LiCl-gel electrolyte (see details in ‘Methods’ and Supplementary Fig. S1). The concentration of electrolytes was measured by molality (mol kg^−1^), which was abbreviated as ‘m’. As shown in Fig. 1a, the relationship between the concentration and ionic conductivity is manifested in a volcanic diagram. At a concentration of 5 m, the WIL electrolyte exhibited the highest ionic conductivity of 170 mS cm^−1^. This was caused by the increase in the number of conductive particles in a unit volume of the solution and the enhancement of the interaction forces among ions. Even when the concentration was increased to 20 m, it still had a high ionic conductivity of 71.2 mS cm^−1^. Further, when a gel electrolyte was formed, the ionic conductivity remained almost unchanged (69.5 mS cm^−1^). To shed light on the electrolyte structure, the difference between lowly and highly concentrated LiCl electrolytes was studied by using Raman spectroscopy (Fig. 1b) and molecular dynamics (MD) simulations (Fig. 1c and d). From the Raman spectra (Fig. 1b), it could be inferred that the O–H stretching vibration modes gave rise to a broad Raman band in the range of 3000–3750 cm^−1^ in pure water. When the concentration of LiCl was increased, the broad band of water clusters at 3250 cm^−1^ gradually disappeared. This was attributed to the participation of water molecules in coordination with Li^+^ and the significant decrease in water clusters. The highly concentrated 20 m LiCl electrolyte showed only a sharp peak at 3450 cm^−1^, indicating coordination of most water molecules with Li^+^ ions. The movement of water molecules was suppressed by this ordered electrolyte structure, consequently reducing their activity [11]. Notably, in the 1 m LiCl electrolyte (Fig. 1c), Li^+^ ions are solvated by four water molecules and well separated from Cl^−^, and lots of free water molecules tend to form a network, corresponding to the broad peak in the Raman spectra. In sharp contrast, most of water molecules in the 20 m LiCl electrolyte are coordinated with Li^+^ ions via oxygen atoms and exhibit negligible hydrogen bonding (Fig. 1d). The Cl^−^ ions also coordinate partially with Li^+^ ions due to the limited amount of water, resulting in strong cation–anion interaction, corresponding to the disappearance of the wide Raman band and enhanced peak at 3450 cm^−1^ (Fig. 1b). In such a situation, most water molecules and some of the anions are included in the Li-ion solvation shells, contributing to the diminished activity of water molecules and enhanced stability of the electrolyte.

Due to the high content of LiCl with a strong Li^+^ solvation effect, the freezing point of the 20 m LiCl-gel electrolyte was decreased to −57°C (Fig. 1e), which is much lower than that of the 1 m LiCl-gel electrolyte (−26°C). Moreover, the electrochemical stability window (ESW) of the 20 m LiCl electrolyte expanded to ∼2.7 V (−1.5 to 1.2 V vs. Ag/AgCl, Fig. 1f) due to the negative transfer of the cathodic limit potentials, while being only ∼1.9 V for the 1 m LiCl electrolyte. As shown in Fig. 1g, the voltage window of the MXene electrode was broadened to as high as 1.8 V without significant water decomposition in the 20 m LiCl electrolyte. In its 1 m counterpart, both the hydrogen evolution reaction below −1.0 V and the oxidative reaction above 0.2 V occurred, thereby limiting the voltage window of the MXene.

### Electrochemical mechanism of MXene

**Figure 2.**
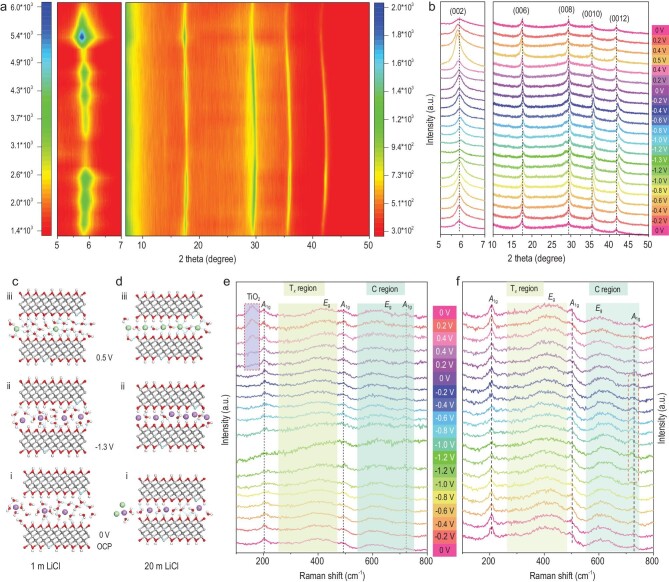
Electrochemical *ex situ* X-ray diffraction (XRD) patterns and *in situ* Raman spectra of MXene. (a) Contour plot and (b) corresponding *ex situ* XRD patterns of MXene in 20 m LiCl electrolyte. Schematic of the energy-storage mechanism of MXene in (c) 1 m and (d) 20 m LiCl electrolyte. *In situ* Raman spectra of MXene in (e) 1 m and (f) 20 m LiCl electrolyte.

The colloidal solution of MXene Ti_3_C_2_T*_x_* flakes was synthesized by the selective etching of Ti_3_AlC_2_ using LiF/HCl (Supplementary Fig. S2) [21]. The typical MXene nanosheets showed micrometer size in the lateral dimension (Supplementary Fig. S2a), a hexagonal crystalline structure (Supplementary Fig. S2b) and a thickness of ∼2.2 nm (Supplementary Fig. S2c). The specific surface area and pore volume of MXene nanosheets were found to be 20.6 m^2^ g^−1^ and 0.05 cm^3^ g^−1^, respectively (Supplementary Fig. S3). The electrical conductivity of the microelectrode film of MXene-MSC-3.2 was measured to be ∼3850 S cm^−1^ (Supplementary Fig. S4). After MXene was placed in LiCl electrolytes, the (000*l*) peaks position of Ti_3_C_2_T*_x_* shifted to a lower Bragg angle (Supplementary Fig. S5 and Fig. 2a and b). The spontaneous access of electrolyte/solvated ions and water molecules to the interlayer space of Ti_3_C_2_T*_x_* caused the initial increase in the interlayer distance (Fig. 2c-i and d-i) [9,22]. The spacing of the (002) peak was increased from 14.65 to 16.63 Å in the 1 m LiCl electrolyte, corresponding to a 3.96 Å increase in the *c*-lattice parameter while an increase of only 0.84 Å was observed in the 20 m LiCl electrolyte due to limited free water molecules. Under negative voltage, the diffraction peaks were shifted to a higher angle (Fig. 2a and b, and Supplementary Fig. S6). From 0 to –1.3 V, the *c*-lattice parameter decreased by 1.2 Å in the 1 m LiCl electrolyte, whereas a smaller shrinkage (0.2 Å) was observed in the 20 m LiCl electrolyte. This shrinkage was attributed to the electrostatic attraction between the Li^+^ and MXene layers (Fig. 2c-ii and d-ii). The diffraction peak from –1.3 to 0 V could not be recovered reversibly in the 1 m LiCl electrolyte. Under the positive voltage from 0 to 0.5 V, the diffraction peak was shifted to a small angle with the expansion of the *c*-lattice parameter in the 20 m LiCl electrolyte, signifying that Cl^−^ ions were capable of facile intercalation and thus increased the Ti_3_C_2_T*_x_* interlayer distance (Fig. 2d-iii). Consequently, it triggered an obvious oxidation peak in the cyclic voltammetry (CV) curve at ∼0.25 V (Fig. 1g). In the 20 m LiCl electrolyte, when the voltage returned to the initial conditions, these diffraction peaks were reversely shifted back. This phenomenon was not observed in the 1 m LiCl electrolyte (Supplementary Fig. S6). It is speculated that the high content of LiCl promoted anion intercalation to regulate kinetically interfacial reactions, inhibiting the oxidation of MXene and broadening the voltage window of MXene in aqueous electrolytes. Moreover, the intercalation/extraction reversibility of ions between MXene interlayers in high-concentration electrolytes endowed MXene with excellent stability during the electrochemical cycle and durability. These observations verified the intercalation pseudocapacitance mechanism of MXene in the 20 m LiCl electrolyte, which ultimately contributed to the higher energy density than the double-layer capacitance.

*In* *situ* Raman spectroscopy was further carried out during CV measurement (Fig. 2e and f). The Raman modes of the as-prepared Ti_3_C_2_T*_x_* at ∼200 cm^−1^ for *A*_1g_ (Ti, O, C) and ∼730 cm^−1^ for *A*_1g_ (C) represent the symmetric out-of-plane vibrations of the Ti, C and surface groups, respectively, while the region of 230–470 cm^−1^ denotes the *E*_g_ group vibrations solely affected by surface atoms, containing in-plane (shear) modes of terminal groups attached to titanium atoms [23,24]. The region of 580–730 cm^−1^ is due to the *E*_g_ and *A*_1g_ vibrations of C atoms. As shown in Supplementary Fig. S7, it was found that the spontaneous intercalation of Li^+^ ions into the interlayer of Ti_3_C_2_T*_x_* enhanced the *A*_1g_ (OH) vibrations of the surface groups at ∼500 cm^−1^ [25]. Interestingly, the *A*_1g_ (C) peak shifted to ∼720 cm^−1^ when the interlayer spacing became smaller (0 → –1.3 V). Conversely, the *A*_1g_ (C) peak shifted oppositely (–1.3 → 0 V). The evolution of the Raman modes remained reversible throughout the electrochemical cycle in the 20 m LiCl electrolyte. However, in the 1 m LiCl electrolyte, a new *E*_g_ vibrational mode of TiO_2_ appeared at 160 cm^−1^ as the operating voltage was increased from 0 to 0.5 V, which existed even when the voltage returned to the initial value, demonstrative of the irreversible oxidization of MXene. However, the Raman modes of  TiO_2_ were not observed in the 20 m LiCl electrolyte. This correlation suggests that the oxidation of MXene is inhibited in the high-concentration LiCl electrolyte, thus broadening the voltage window of MXene in aqueous electrolytes.

### Electrochemical characterization of MXene-MSCs

**Figure 3.**
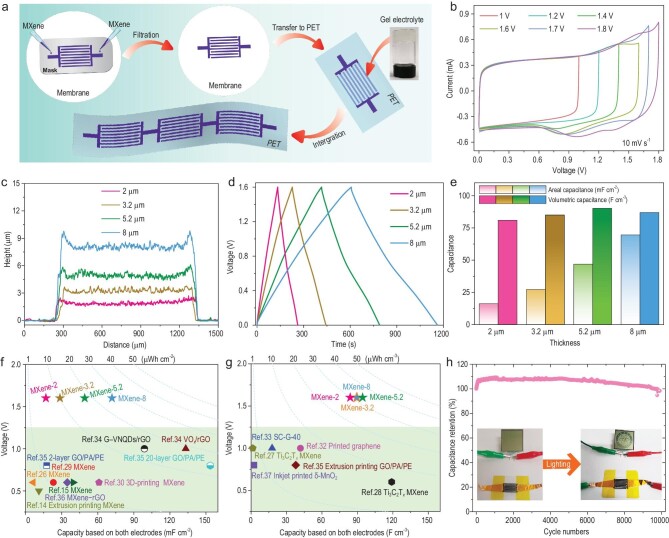
Fabrication schematic and electrochemical performances of MXene-MSCs in 20 m LiCl-gel electrolyte. (a) Fabrication and (b) CV curves at different potential windows of MXene-MSCs. (c) Height profiles, (d) GCD profiles and (e) corresponding areal capacitance and volumetric capacitance of MXene-MSCs with different microelectrode thicknesses. (f) and (g) Comparison of the voltage, capacitance and energy density of MXene-MSCs with various previously reported MSCs: (f) areal capacitance and energy density and (g) volumetric capacitance and energy density. (h) Long-term cycling stability of MXene-MSCs. The insets are the photographs of our institute ‘DICP’ logo powered by MXene-MSC.

The fabrication steps of MXene-MSCs are schematically illustrated in Fig. 3a. The electrochemical measurements showed that the stable voltage window of MXene-MSCs could be broadened to 1.6 V in the 20 m LiCl-gel electrolyte; however, the obvious polarization occurred in the 1 m and 5 m LiCl gel electrolytes (Fig. 3b and Supplementary Fig. S8). MXene-MSCs with different electrode thicknesses of 2, 3.2, 5.2 and 8 μm were denoted as MXene-MSC-*x* (*x* represents the thickness, Fig. 3c). The surface profile displayed uniformly flat microelectrode fingers (Supplementary Fig. S9). The lateral section of the microelectrode finger clearly unveiled the deposited MXene layers consisting of mutually overlapping few-layered flakes (Supplementary Fig. S10), with a layered structure favoring the rapid transport of ions. The thickness-dependent performance is further demonstrated in Fig. 3d and e, and Supplementary Figs S11–S15. The nearly linear galvanostatic charge and discharge (GCD) profiles and vertical electrochemical impedance spectroscopy (EIS) curves of MXene-MSCs in the low-frequency region (Fig. 3d and Supplementary Figs S11–S14) confirmed the pseudocapacitance feature of MXene. The areal capacitance of MXene-MSCs increased linearly from 16 to 70 mF cm^−2^ at 0.2 mA cm^−2^ as the thickness increased (Fig. 3e). Notably, the volumetric capacitances remained close to 86 F cm^−3^ for all measured thicknesses. These test results show that the microelectrode thicknesses have an obvious effect on the areal capacitance, but not so much on the volumetric capacitance. It is well accepted that the optimal electrode thickness not only increases the capacitance, but also reduces the electron and ion diffusion resistance. Thereby, the comprehensive electrochemical results (Supplementary Figs S15–S18) demonstrate that MXene-MSC-3.2 has an excellent rate performance. Notably, the voltage, areal/volumetric capacitance and energy density of MXene-MSCs are superior to most previously reported symmetric MSC devices (Fig. 3f and g) [1,26–37]. Since our MXene-MSCs exhibited a high output voltage of 1.6 V, by tuning to the suitable electrode thickness, MXene-MSC-3.2 exhibited the highest areal and volumetric capacitance of 28.5 mF cm^−2^ and 89.2 F cm^−3^ at 0.1 mA cm^−2^. By virtue of the enhanced voltage window, high areal and volumetric energy density of 10.1 μWh cm^−2^ and 31.7 mWh cm^−3^ could be achieved at the power density of 80 μW cm^−2^ and 250 mW cm^−3^, respectively, which surpassed previously reported MXene-based or other symmetric MSCs (Supplementary Table S3). In addition, MXene-MSC-3.2 also showed excellent cycling stability with almost no capacity decay over 10 000 cycles and could easily power a liquid crystal display (inset: the optical image of the Dalian Institute of Chemical Physics (DICP) logo, Fig. 3h).

**Figure 4.**
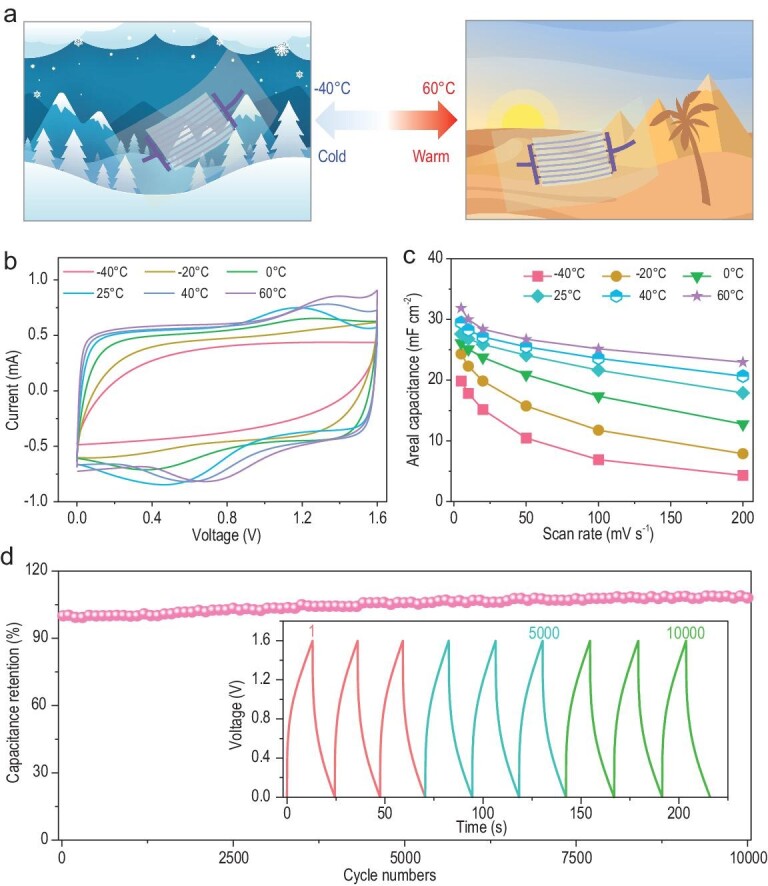
Schematic illustration and characterization of wide-temperature MXene-MSC-3.2. (a) Schematic illustration of MXene-MSC-3.2 in the cold and warm. (b) CV curves and (c) areal capacitances obtained at different operating temperatures. (d) Cycling stability under –40°C; the inset shows GCD profiles during charge/discharge cycles.

To demonstrate optimal performance under harsh environmental conditions, the electrochemical performance of MXene-MSC-3.2 from –40 to 60°C was evaluated (Fig. 4a and Supplementary Figs S19–S21). With the ultralow freezing point and heat-tolerant features of WIL gel electrolyte, MXene-MSC-3.2 was able to operate in a wide temperature range from −40 to 60°C. It was observed that the capacitance and rate performance of MXene-MSC-3.2 were improved with the increase in temperature (Fig. 4b and Supplementary Fig. S21). It is worth noting that MXene-MSC-3.2 exhibited an areal capacitance of 19.9 mF cm^−2^ at 5 mV s^−1^ (Fig. 4c and Supplementary Fig. S19) and 20.1 mF cm^−2^ at 0.1 mA cm^−2^ (Supplementary Figs S20 and S21) at –40°C, corresponding to a retention of 72.1% and 70.3% compared to the capacitance at 25°C. Even at such a low temperature, the capacitance increased slowly before stabilizing without any attenuation during 10 000 cycles at 1 mA cm^−2^ (Fig. 4d), exhibiting great potential for MSCs to operate under harsh environmental conditions.

**Figure 5.**
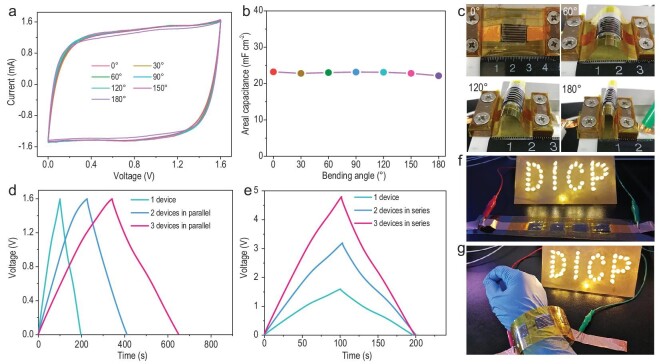
Flexibility and integration characterization of MXene-MSC-3.2. (a) CV curves, (b) capacitance retention and (c) corresponding optical images at various bending angles. GCD profiles of MXene-MSC-3.2 in (d) parallel and (e) series from one to three cells obtained at 0.5 mA cm^−2^. Optical images of the letter ‘DICP’, made up of LED lights, illuminated by serially connected MXene-MSC-3.2 in (f) flatting and (g) bending wearable states.

Remarkably, our MXene-MSC-3.2 exhibited excellent flexibility without any fragmentation or delamination from a polyethylene terephthalate (PET) substrate even under severe deformation, e.g. bending and twisting (Supplementary Fig. S22). The MXene-MSC-3.2 exhibited only minor fluctuations in CV curves with variable bending angles (Fig. 5a) and retained ∼98% of the original capacitance (Fig. 5b) even tested at a 180° bent state, indicating the excellent mechanical flexibility and structural stability of the shape-customized devices. The corresponding photographs are displayed in Fig. 5c. Such remarkable flexibility is primarily attributed to the planar geometry and unique lamellar structure of MXene, excellent integrity of interconnections and microelectrodes, and usage of LiCl-gel electrolyte, manifesting significant potentiality for the seamless integration of MXene-MSCs in the emerging field of flexible microelectronics. Moreover, the integrated MXene-MSC-3.2 connected in series or parallel was easily implemented based on a one-by-one transfer strategy (Supplementary Fig. S23) and also demonstrated excellent flexibility. The GCD and CV curves of the integrated MXene-MSC-3.2 parallel devices showed a linear increase in current and capacitance (Fig. 5d and Supplementary Fig. S24), suggestive of outstanding performance uniformity. Meanwhile, the operating voltage of MXene-MSC-3.2 could be further extended from 1.6 to 4.8 V by an in-series connection from one to three cells (Fig. 5e and Supplementary Fig. S24), indicative of tunable voltage output. Furthermore, the four serially connected MXene-MSC-3.2 pack was capable of readily lighting up the four letters of DICP comprising 42 light-emitting diodes (LEDs) (Fig. 5f) and remained lit while wearing it on the wrist (Fig. 5g), signifying the superb potential of MXene-MSCs as a standalone micropower source for safe wearable microelectronics.

## CONCLUSION

In summary, we have developed a cost-effective and inherently safe aqueous WIL salt electrolyte that effectively adjusts the interfacial reactions of MXene electrodes and electrolytes, and inhibits the oxidation of MXene at high anodic potential, endowing MXene-MSCs with a record operating voltage of 1.6 V, ultra high areal/volumetric energy density and excellent environmental adaptability for symmetric devices. Aqueous WIL gel electrolytes provide an important and straightforward direction for the practical implementation of aqueous MSCs with high operation voltage and wide temperature range. This work also clarifies the energy-storage mechanism of MXene-MSCs in WIL electrolytes through *insitu* Raman and *exsitu* XRD investigations, which provides universal guidance to the exploration and design of other high-concentration salt electrolytes and aqueous high-voltage MXene-based MSCs in both symmetric configurations. Therefore, we believe that through further exploration of high-voltage WIL electrolytes and reasonable matching of the positive and negative electrodes, the operating voltage, temperature range and energy density of MXene-based MSCs can be upgraded further, widening the adaptation and applications of wearable and safe microelectronics.

## METHODS

### Preparation of electrolytes

LiCl salt (Aladdin, China) was dissolved into deionized water to prepare aqueous LiCl electrolytes (1, 2, 5, 10, 15 and 20 m). The LiCl-gel electrolytes (1 and 20 m) were prepared by mixing ∼6 wt% SiO_2_ powder (0.007 μm, Sigma-Aldrich) and aqueous LiCl electrolytes.

### Fabrication of symmetric MXene-MSCs

The MXene-MSCs were fabricated by using a vacuum filtration technique for MXene dispersion with an interdigital mask (length: 14 mm, width: 1 mm, interspace: 500 μm), followed by a transfer process. Typically, MXene dispersion was uniformly filtrated through a mask to form an interdigital MXene microelectrode on polyvinylidene fluoride membrane (pore size: 0.2 μm). The thickness of the MXene microelectrode finger could be easily controlled by adjusting the concentration and volume of MXene dispersion. After removing the mask, the as-fabricated microelectrodes were fully transferred on a flexible PET substrate under 20 MPa. Finally, MXene-MSCs were obtained after drop-casting LiCl-gel electrolyte onto the projected area of the interdigital electrodes. Before the test of MSCs, the MSCs were fully packaged with Kapton tape, using conductive copper tape as the wire to connect the microelectrodes with the external circuit.

### Materials characterization

The characterization of the morphology and structure was carried out using a field-emission scanning electron microscope (FESEM, JSM-7800F), a high-resolution transmission electron microscope (HRTEM, JEM-2100), an atomic force microscope (Asylum Research MFP), XRD (X'pert Pro) and Micromeritics autosorb instruments. The *exsitu* XRD (Smartlab, Rigaku) was carried out on the MXene electrode that was charged or discharged at a selected voltage and XRD data were collected in the range of 2θ = 5–50°. The thickness and surface profile mapping were performed using a stylus profiler (Alpha step D-600). Raman spectroscopy was performed using a high-resolution Raman spectrometer (LabRAM-HR800) equipped with a 532 nm excitation laser and a 50× long working distance objective. A three-electrode configuration was used for electrochemical *insitu* Raman measurement, MXene as the working electrode, Ag/AgCl (sat. KCl) as the reference electrode and platinum wire as the counter electrode. *Insitu* Raman spectra were collected during the CV test from open-circuit potential (OCP, 0 V) to low potential, with a range of –1.3 to 0.5 V. Differential scanning calorimetry (DSC) analysis was carried out using a DSC 204 HP analyser under N_2_ atmosphere using a liquid nitrogen cooling system from 60 to −90 °C at 10 °C min^−1^. The ionic conductivities of the electrolytes were measured using a conductivity meter (Mettler-Toledo S230-K seven compact conductivity meter).

### Electrochemical measurement

The electrochemical performances of the MXene electrode, electrolyte and MXene-MSCs were recorded at the electrochemical workstation (CHI 760E). The CV tests were performed at scan rates from 5 to 200 mV s^−1^ and GCDs profiles were obtained with current densities from 0.1 to 10 mA cm^−2^. The ESW of the aqueous LiCl electrolyte was measured using linear sweep voltammetry at a scan rate of 10 mV s^−1^ with a glass carbon sheet as the working electrode, a Ag/AgCl (in saturated KCl) electrode as the reference electrode and activated carbon (YP-50) as the counter electrode. EIS measurements were carried out with frequencies from 0.01 Hz to 100 kHz under an amplitude of 5 mV at the open-circuit potential.

## Supplementary Material

nwac024_Supplemental_File
