# Supplementary material for: Kinetic regulation of MXene with water-in-LiCl electrolyte for high-voltage micro-supercapacitors
Source: Natl Sci Rev. 2022 Feb 23;9(7):nwac024. doi: 10.1093/nsr/nwac024 (PMC9283101; doi:10.1093/nsr/nwac024)
Supplement: nwac024_Supplemental_File [file nwac024_supplemental_file.docx]

**Supplementary Information for**

Kinetic regulation of MXene with water-in-LiCl electrolyte for high-voltage micro-supercapacitors

Yuanyuan Zhu^1,†^, Shuanghao Zheng^1,†^, Pengfei Lu^1^, Jiaxin Ma^1,5^, Pratteek Das^1,5^, Feng Su^1,5^, Hui-Ming Cheng^2,3,^*, Zhong-Shuai Wu^1,4,^*

^1^ State Key Laboratory of Catalysis, Dalian Institute of Chemical Physics, Chinese Academy of Sciences, Dalian 116023, China

^2^ Shenyang National Laboratory for Materials Science, Institute of Metal Research, Chinese Academy of Sciences, Shenyang 110016, China

^3^ Shenzhen International Graduate School, Tsinghua University, Shenzhen 518055, China

^4^ Dalian National Laboratory for Clean Energy, Chinese Academy of Sciences, Dalian 116023, China

^5^ University of Chinese Academy of Sciences, Shijingshan District, Beijing 100049, China

∗Corresponding author. E-mail: [cheng@imr.ac.cn](mailto:cheng@imr.ac.cn); [wuzs@dicp.ac.cn](mailto:wuzs@dicp.ac.cn)

^†^ Equally contributed to this work.

**METHODS**

**Synthesis of Ti_3_C_2_T_x_ MXene**

Ti_3_C_2_T*_x_* MXene was synthesized by etching Ti_3_AlC_2_ using LiF/HCl etchant [1]. Typically, 0.5 g LiF was dissolved into 9 M HCl (10 mL). Then, 0.5 g Ti_3_AlC_2_ was slowly added to the above solution with continuous stirring. After stirring at 35 ^o^C for 24 h, the etched product was centrifuged several times with deionized water (3500 rpm, 5 min) until pH = 6. The obtained multi-layer Ti_3_C_2_T*_x_* precipitation with a yield of ~90% was mixed with 20 mL deionized water and then shaken vigorously by the vortex machine. Subsequently, the resultant dispersion was centrifuged at 3500 rpm for 1 h and the supernatant was collected for further usage. After being fully dispersed in deionized water, Ti_3_C_2_T*_x_* MXene dispersion (0.5 - 1 mg mL^−1^) was obtained for the preparation of MSCs.

**Molecular dynamics simulations**

To investigate the impact of electrolyte ions on the distributions and Raman activities of the LiCl solutions, two kinds of LiCl aqueous systems were constructed by the Amorphous Cell tool. In the dilute solution, one pair of Li^+^/Cl^−^ ions was mixed with 55 water molecules, while for the concentrated solution, 20 pairs of Li^+^/Cl^−^ ions and 55 water molecules were included. All the simulations were conducted using Materials Studio 8.0 packages. Based on the COMPASS force field, an *ab initio* force field which contains 9-6 Lennard-Jones (LJ-9-6) potential and Coulomb potential, the non-bonding interactions among water molecules and Li^+^/Cl^−^ ions were well described. Under the canonical ensemble (NVT), the energy minimization step was firstly conducted by the steepest descent, conjugate gradient, and quasi-Newton methods to optimize the initial configuration. Second, based on the optimized configurations, a 1 ns molecular dynamic simulation was conducted to bring the system into equilibrium, and then another 1 ns simulation was performed for data collection. In the whole simulation process, the simulation time step was selected as 1 fs and the atomic coordinate and system energy were saved every 10 ps. The van der Waals and Coulomb forces were calculated based on the atom-based addition and Ewald summation methods, respectively. The truncation radius of van der Waals potential was 1.2 nm, the accuracy of Coulomb potential was 10^−3^ kcal mol^−1^. In addition, the whole system was maintained at 298 K with Nose thermostat.

**Calculations**

The capacitance (*C_CV_*) is evaluated from the CV curves based on Equation S1:

*C_CV_* =$\frac{1}{\nu\times\Delta V}\int I(V)dV$ (Equation S1)

The capacitance (*C_GCD_*) is evaluated from the GCD curves based on Equation S2:

*C_GCD_* =$\frac{I\times\Delta t}{\Delta V}$ (Equation S2)

Where *ν* (V s^−1^), *∆V* (V), *I* (A), and *∆t* (s) represent geometry area, scan rate, potential window, current, and discharge time, respectively.

Areal capacitance (*C_A_*), volumetric capacitance (*C_V_*), and gravimetric capacitance (*C_g_*) of MSCs are calculated based on the total area (*A*, cm^−2^), volume (*V*, cm^−3^), and mass (*m*, g) of two electrodes according to Equation S3-5:

*C_A_* = *C*/*A* (Equation S3)

*C_V_* = *C*/*V* (Equation S4)

*C_g_* = *C*/*m* (Equation S5)

The areal energy density (*E_A_*) and power density (*P_A_*) and volumetric energy density (*E_V_*) and power density (*P_V_*) of MSC are calculated from the GCD curves according to Equations S5-8:

*E_A_* =$\frac{1}{2\times3600}$*C_A_* $\times\Delta V$^2^ (Equation S5)

*P_A_* = 3600 *E_A_*/*∆t* (Equation S6)

*E_V_* =$\frac{1}{2\times3600}$*C_V_* $\times\Delta V$^2^ (Equation S7)

*P_V_* = 3600 *E_V_*/*∆t* (Equation S8)


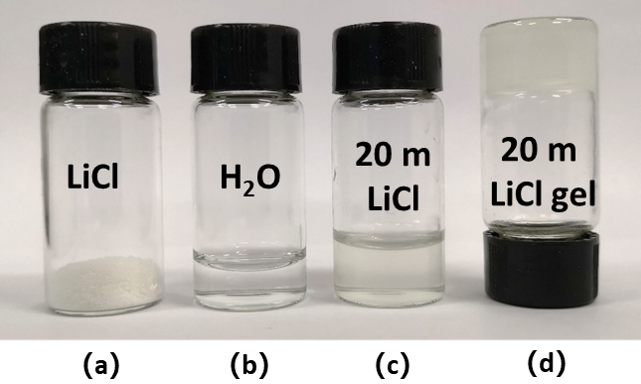


**Figure S1.** Stoichiometric amounts of LiCl (a) and water (b) used to prepare 20 m LiCl (c) and 20 m LiCl-gel (d) electrolytes at room temperature.

**
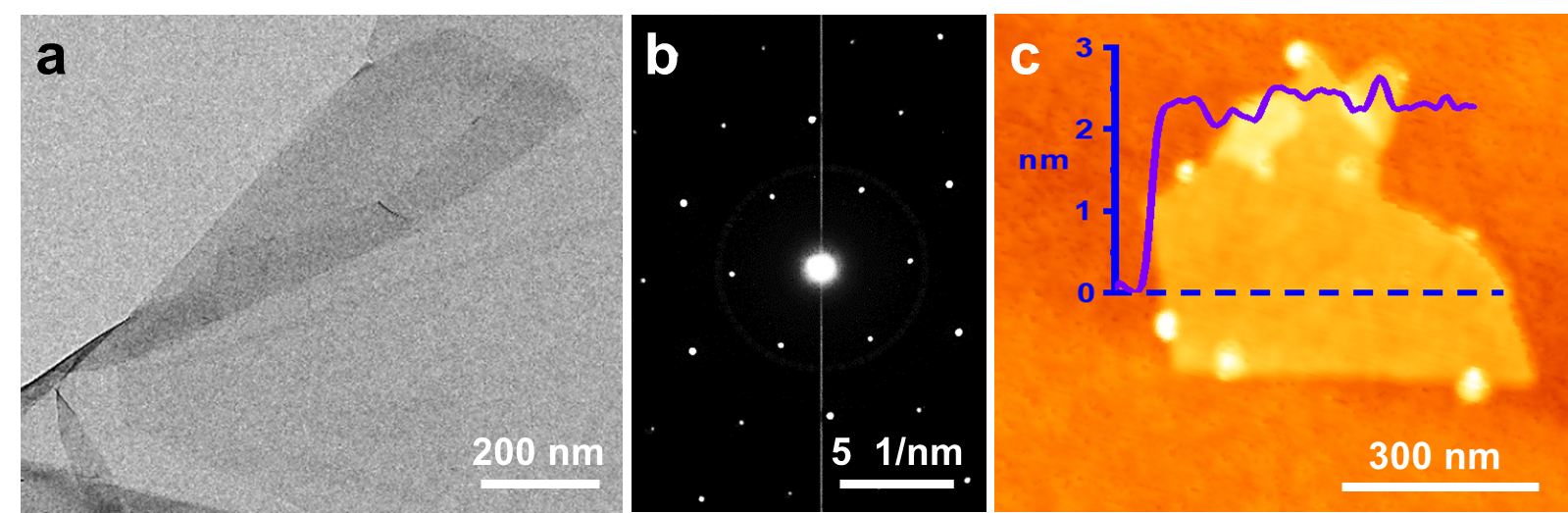
**

**Figure S2.** Morphology and structure characterization of as-prepared 2D Ti_3_C_2_T*_x_* MXene. (a) TEM image, (b) SAED image, and (c) AFM image of Ti_3_C_2_T*_x_* MXene nanosheets. The inset of (c) is the height profile. The MXene nanosheets show the typical micron-sized lateral dimensions and obvious wrinkles, the single set of diffraction spots with 6-fold symmetry with hexagonal crystalline structures, and a single-layer thickness of ~2.2 nm.


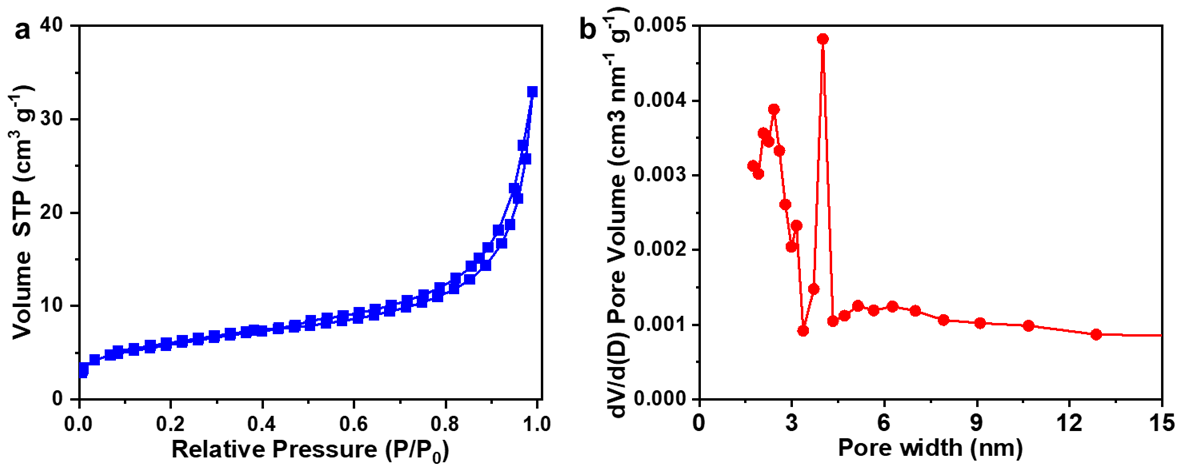


**Figure S3.** (a) N_2_ adsorption and desorption isotherm and (b) corresponding pore size distribution of Ti_3_C_2_T*_x_* MXene nanosheets. The Brunauer-Emmett-Teller surface area and pore volume were calculated to be 20 m^2^ g^−1^ and 0.05 cm^3^ g^−1^, respectively. The pore size distribution curve presented centered pore size at 2.4 nm and 4.0 nm, respectively.





**Figure S4.** Electrical conductivities of MXene microelectrode film with a thickness of ~3.2 μm.


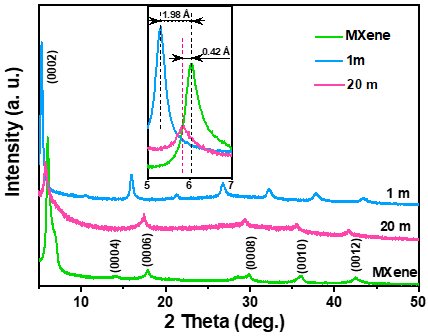


**Figure S5.** Comparison of XRD patterns for Ti_3_C_2_T*_x_* MXene. Ti_3_C_2_T*_x_* MXene placed in 1 m and 20 m LiCl electrolytes compared to the initial Ti_3_C_2_T*_x_* MXene, respectively. The spacing of (0002) peaks of Ti_3_C_2_T*_x_* MXene increased from 14.65 Å to 16.63 Å in 1 m LiCl electrolyte, corresponding to a 3.96 Å increase of the c-lattice parameter while an increase of only 0.84 Å was observed in 20 m LiCl electrolyte due to limited free water molecules.


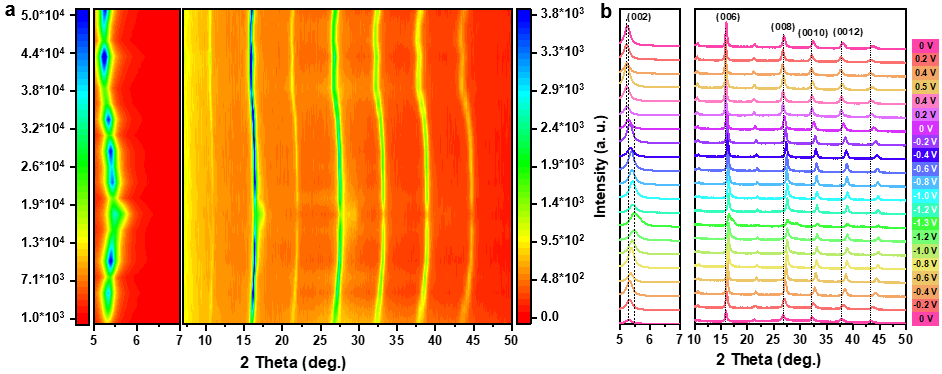


**Figure S6.** Electrochemical *ex-situ* XRD patterns of Ti_3_C_2_T*_x_* MXene. (a) Contour plot and (b) corresponding electrochemical *ex-situ* XRD patterns of Ti_3_C_2_T*_x_* MXene in 1 m LiCl electrolyte during an electrochemical cycle.


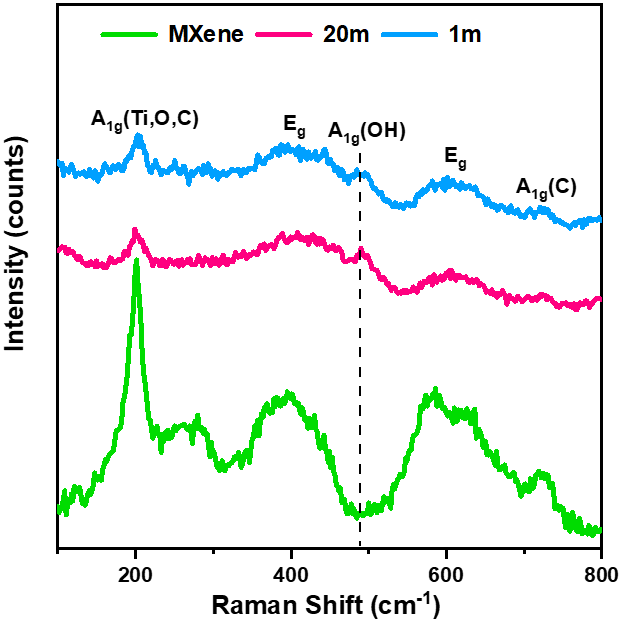


**Figure S7.** Comparison of Raman spectra for Ti_3_C_2_T*_x_* MXene. MXene was placed in 1 m and 20 m LiCl electrolytes compared to the initial MXene, respectively. The spontaneous intercalation of ions into the interlayer of Ti_3_C_2_T*_x_* and the influence of electrolyte weaken the *A*_1g_ (Ti, O, C), *E*_g_, and *A*_1g_ (C) vibrations of the surface groups in 1 m and 20 m LiCl electrolytes.





**Figure S8.** Electrochemical stability windows of MXene-MSCs. The CV curves of MXene-MSCs tested in 1 m, 5 m, and 20 m LiCl-gel electrolytes, respectively (scan rate: 5 mV s^–1^).


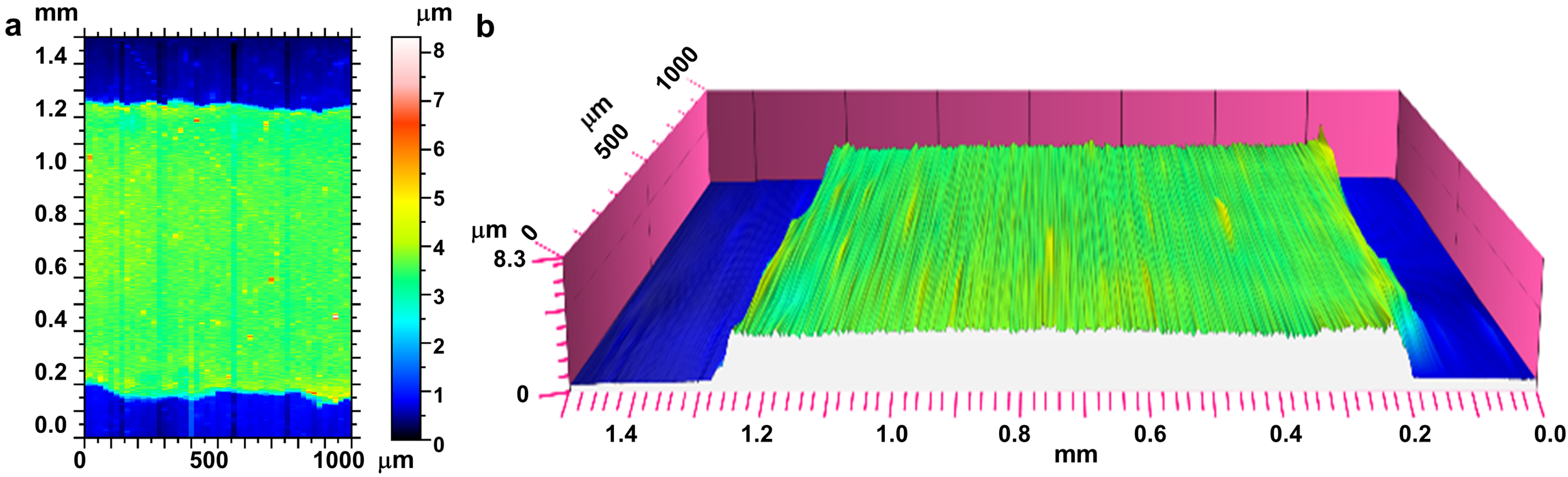


**Figure S9.** (a) 2D pseudo-colour view and (b) 3D view of MXene microelectrode finger with an average thickness of ~3.2 μm on PET substrate.


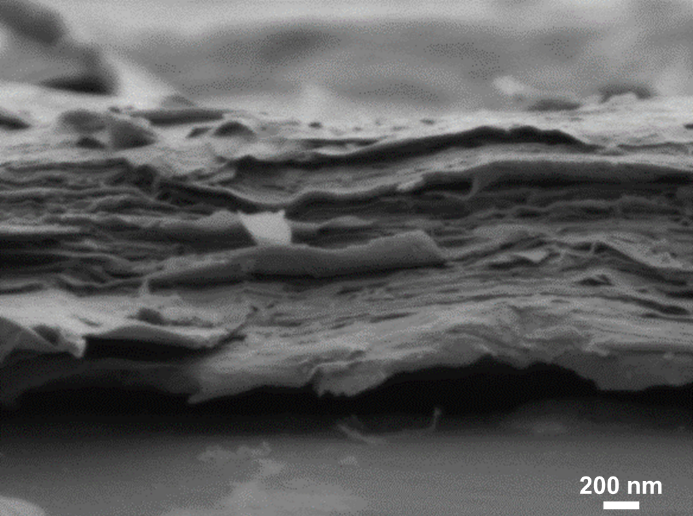


**Figure S10.** SEM image of microelectrode finger for MXene-MSCs. A cross-sectional SEM image of a microelectrode finger for MXene-MSCs shows the layered architecture of the microelectrode constructed by restacking exfoliated 2D Ti_3_C_2_T*_x_* MXene nanosheets.


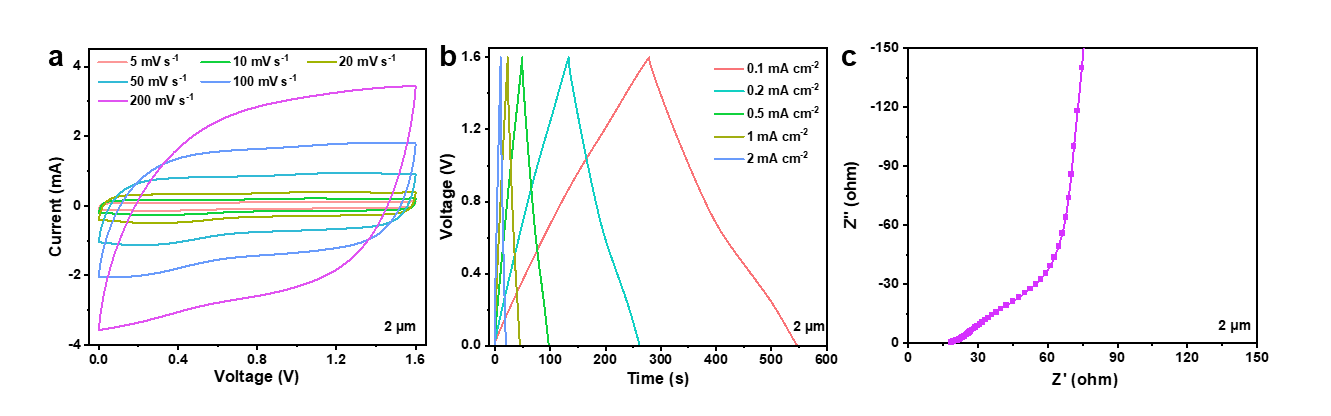


**Figure S11.** Electrochemical performance of MXene-MSC-2. (a) CV curves of MXene-MSC-2. The scan rate varies from 5 to 200 mV s^−1^. (b) GCD curves of MXene-MSC-2. The current density varies from 0.1 to 2 mA cm^−2^. The CV shapes maintain a quasi-rectangular shape and GCD curves are nearly linear, indicating pseudo-capacitance behaviors. (c) The Nyquist plot and fitted equivalent circuit of MXene-MSC-2. The equivalent series resistance (ESR) of 18 Ω and nearly vertical slope in the low-frequency range indicate the strong capacitive behavior.


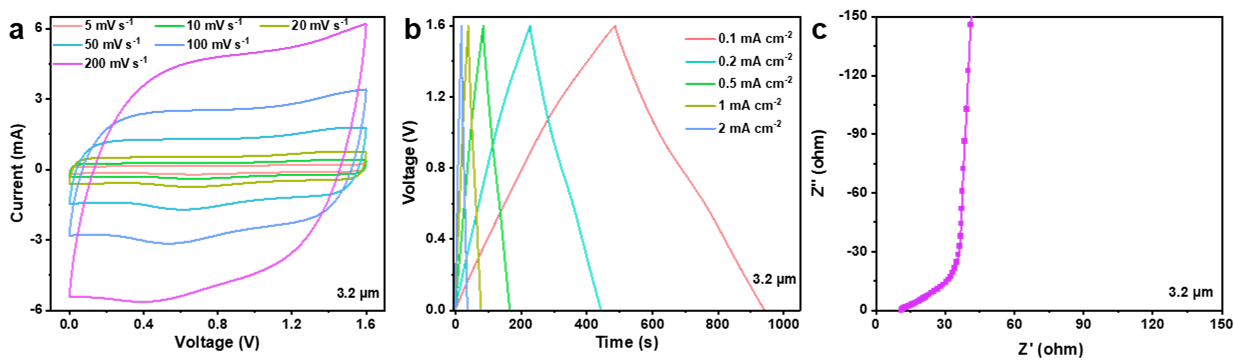


**Figure S12.** Electrochemical performance of MXene-MSC-3.2. (a) CV curves of MXene-MSC-3.2. The scan rate varies from 5 to 200 mV s^−1^. (b) GCD curves of MXene-MSC-3.2. The current density varies from 0.1 to 2 mA cm^−2^. The shape of CV and GCD curves without obvious distortion is observed with a gradual increase of the scan rate and current density, suggesting the good rate capability. (c) The Nyquist plot and fitted equivalent circuit of MXene-MSC-3.2. The lower ESR (11 Ω), charge transfer resistance, and diffusion resistance are the chief reasons for the excellent rate performance.


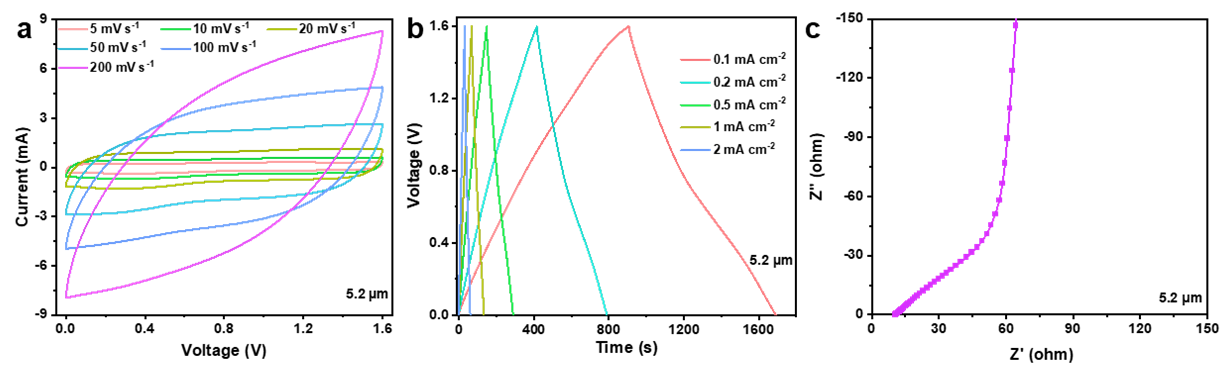


**Figure S13.** Electrochemical performance of MXene-MSC-5.2. (a) CV curves of MXene-MSC-5.2. The scan rate varies from 5 to 200 mV s^−1^. (b) GCD curves of MXene-MSC-5.2. The current density varies from 0.1 to 2 mA cm^−2^. (c) The Nyquist plot and fitted equivalent circuit of MXene-MSC-5.2. When the thickness of the electrode increases to 5.2 μm, the CV curves of MXene-MSC-5.2 show only a slight deformation at the scanning rate exceeding 100 mV s^−1^. Because of the slight increase of charge transfer resistance, the transport of ions is limited.


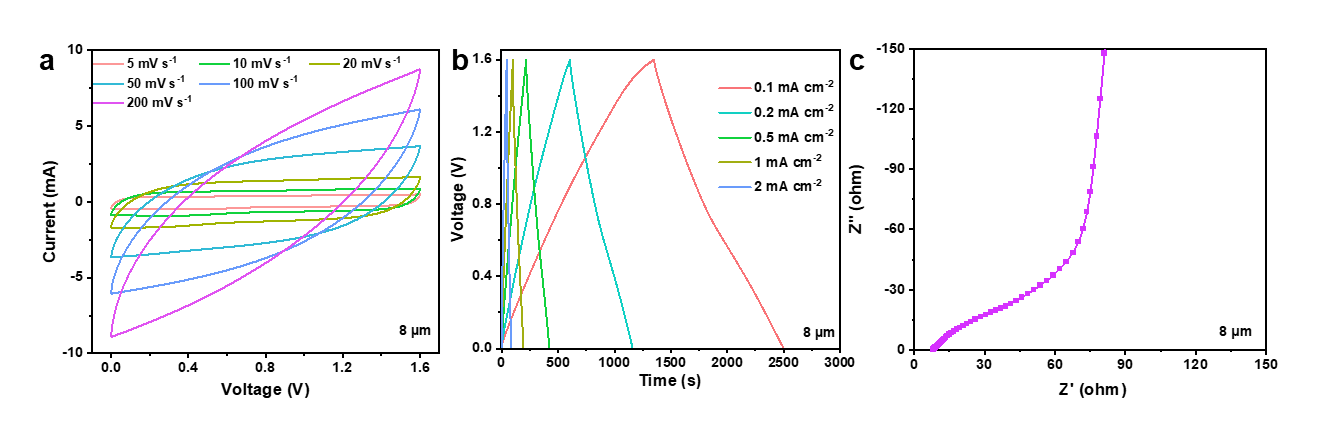


**Figure S14.** Electrochemical performance of MXene-MSC-8. (a) CV curves of MXene-MSC-8. The scan rate varies from 5 to 200 mV s^−1^. (b) GCD curves of MXene-MSC-8. The current density varies from 0.1 to 2 mA cm^−2^. (c) The Nyquist plot and fitted equivalent circuit of MXene-MSC-8. The increase of charge transfer resistance hinders the rapid transport of electrolyte ions to some extent due to the increase of electrode thickness.


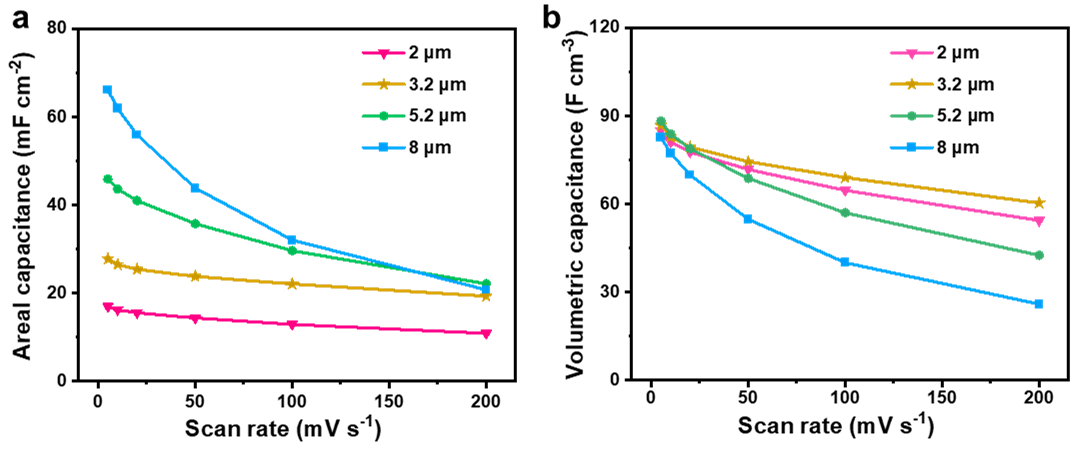


**Figure S15.** Variation of (a) areal capacitance and (b) volumetric capacitance with different scan rates for MXene-MSCs with different microelectrode thicknesses. For the MXene-MSC-3.2, the high areal capacitance (19 mF cm^−2^) and volumetric capacitance (60 F cm^−3^) are maintained at a scan rate of up to 200 mV s^−1^.





**Figure S16.** Gravimetric capacitance of MXene-MSC-3.2 obtained at different scan rates.





**Figure S17.** Self-discharge curve of MXene-MSCs in 20 m LiCl-gel electrolyte. After charging to 1.6 V and holding for 400 s, the decay of the open circuit voltage was evaluated during 10 h. The trend of sharp decline followed by flattening out can be clearly observed for the self-discharge curve. The open circuit voltage of MXene-MSC fell off from 1.6 V to 0.7 V within 10 h. It is demonstrated that MXene-MSCs have slow self-discharge characteristics in 20 m LiCl-gel electrolyte.


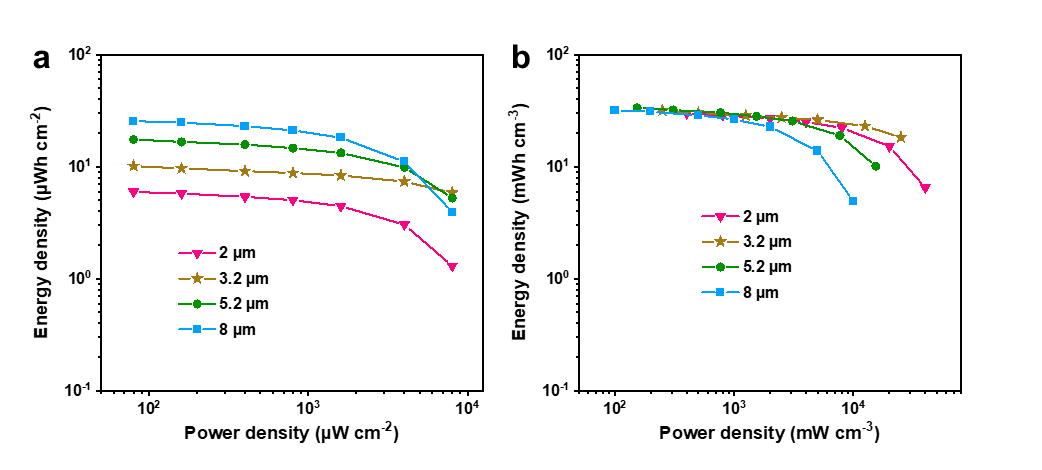


**Figure S18.** Ragone plots of (a) areal and (b) volumetric energy density and power density of MXene-MSCs with different microelectrode thicknesses. With the increase in thickness, the energy density of MXene-MSCs increases gradually, and the highest areal and volumetric energy density can reach 25.5 μWh cm^−2^ and 33.6 mWh cm^−3^ at the power density of 80 μW cm^−2^ and 154 mW cm^−3^, respectively. The volumetric energy density of MXene-MSC-3.2 is still as high as 18.2 mWh cm^−3^ at a high power density of 25,000 mW cm^−3^.

**
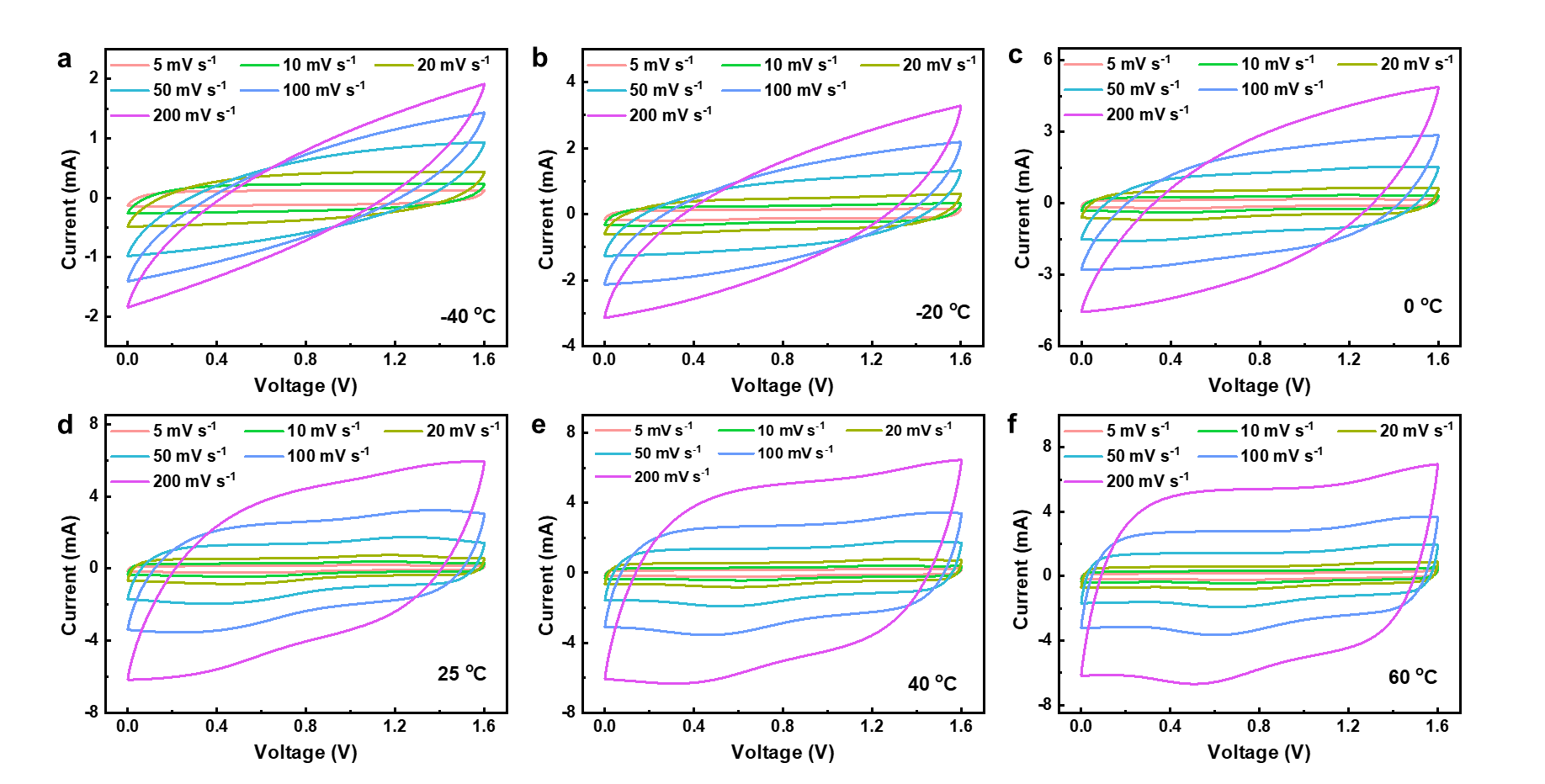
**

**Figure S19.** CV curves of MXene-MSCs at various temperatures. (a) −40 ℃, (b) −20 ℃, (c) 0 ℃, (d) 25 ℃, (e) 40 ℃ and (f) 60 ℃. The shapes of CV curves change with the changes of temperature. CV curves become more rectangular and the area of CV curve also increases, due to the rapid transfer characteristic of the electrolyte ion with the increase of temperature.

**
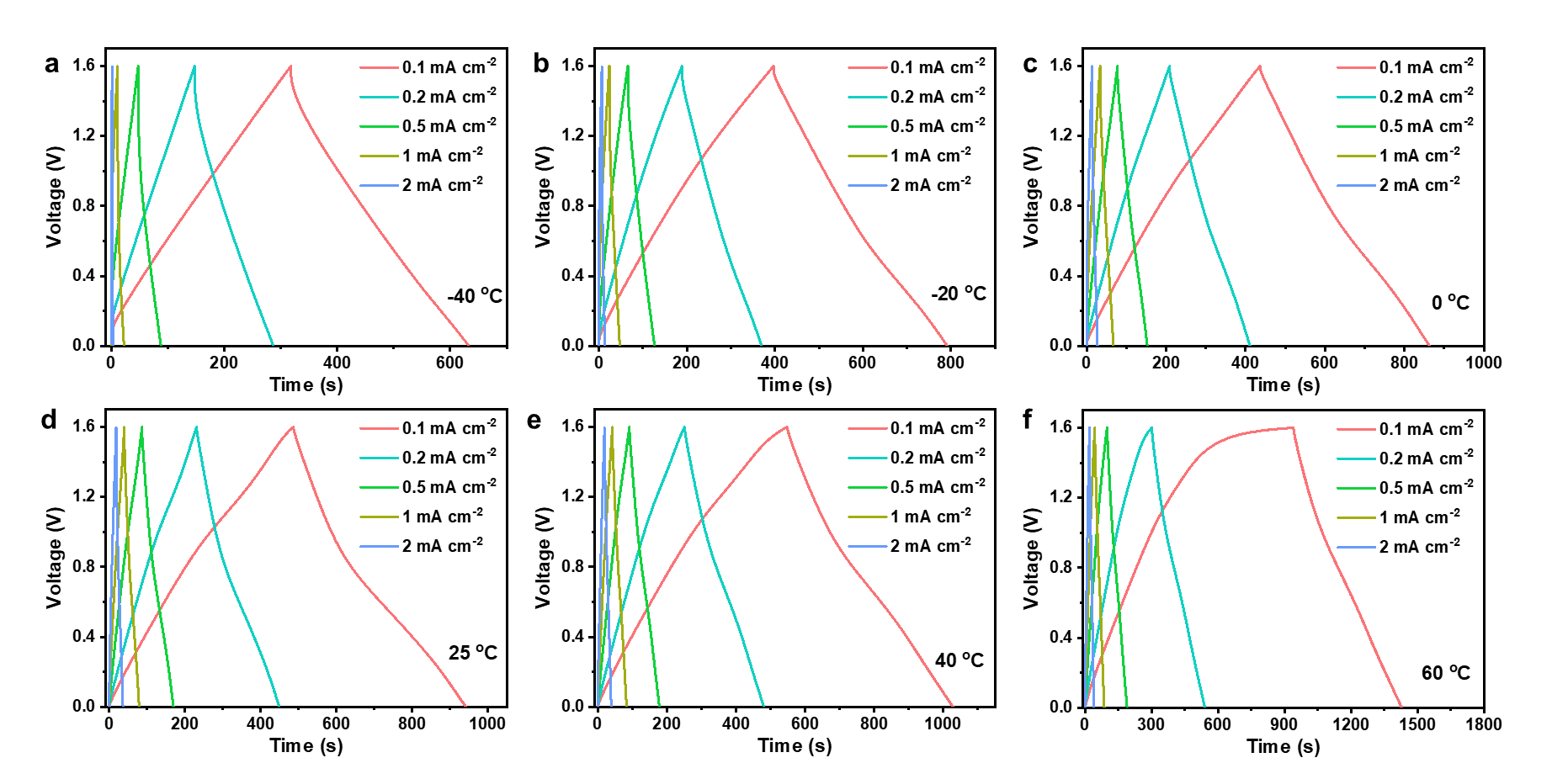
**

**Figure S20.** GCD curves of MXene-MSCs at various temperatures. (a) −40 ℃, (b) −20 ℃, (c) 0 ℃, (d) 25 ℃, (e) 40 ℃ and (f) 60 ℃. Even at −40 ℃, GCD curves of MXene-MSCs still retain symmetric shapes, suggesting high Coulombic efficiencies. Higher temperature makes the discharge time grow longer, implying the increase of the capacitance.


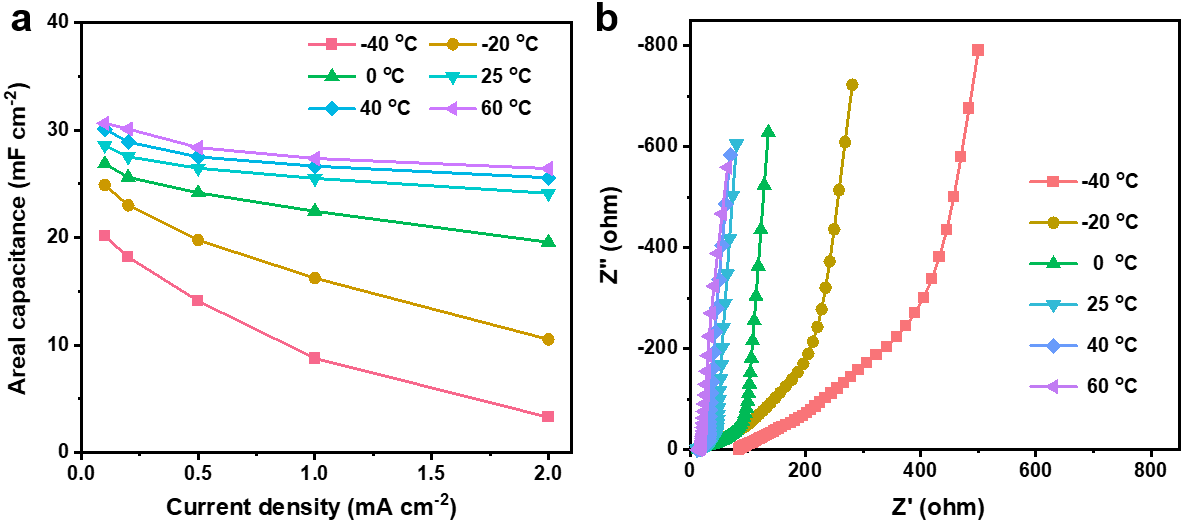


**Figure S21.** Temperature-dependent investigation of MXene-MSCs. (a) Variation of areal capacitance with various current densities. (b) Nyquist plots of MXene-MSCs at various operating temperatures. MXene-MSCs exhibit high areal capacitance of 30.6 mF cm^−2^ at 0.1 mA cm^−2^ under 60 °C, even at a high current density of 2 mA cm^−2^, the capacitance still retains 86% (26.4 mF cm^−2^). The areal capacitance of MXene-MSCs slightly increases above 25 °C, which is quite different from those in cold conditions. The variation of impedance with working temperature is analyzed by EIS. With the increase of temperature, the impedance spectra shift to the left and decrease to achieve better electrochemical performance. Above 25 °C, the charge transfer resistances decrease slightly and the ion diffusion kinetics accelerates. Below 25 °C, the charge transfer resistances increase rapidly, which seriously limits the ion diffusion kinetics, resulting in obvious differences in electrochemical performance at high and low temperatures.

**
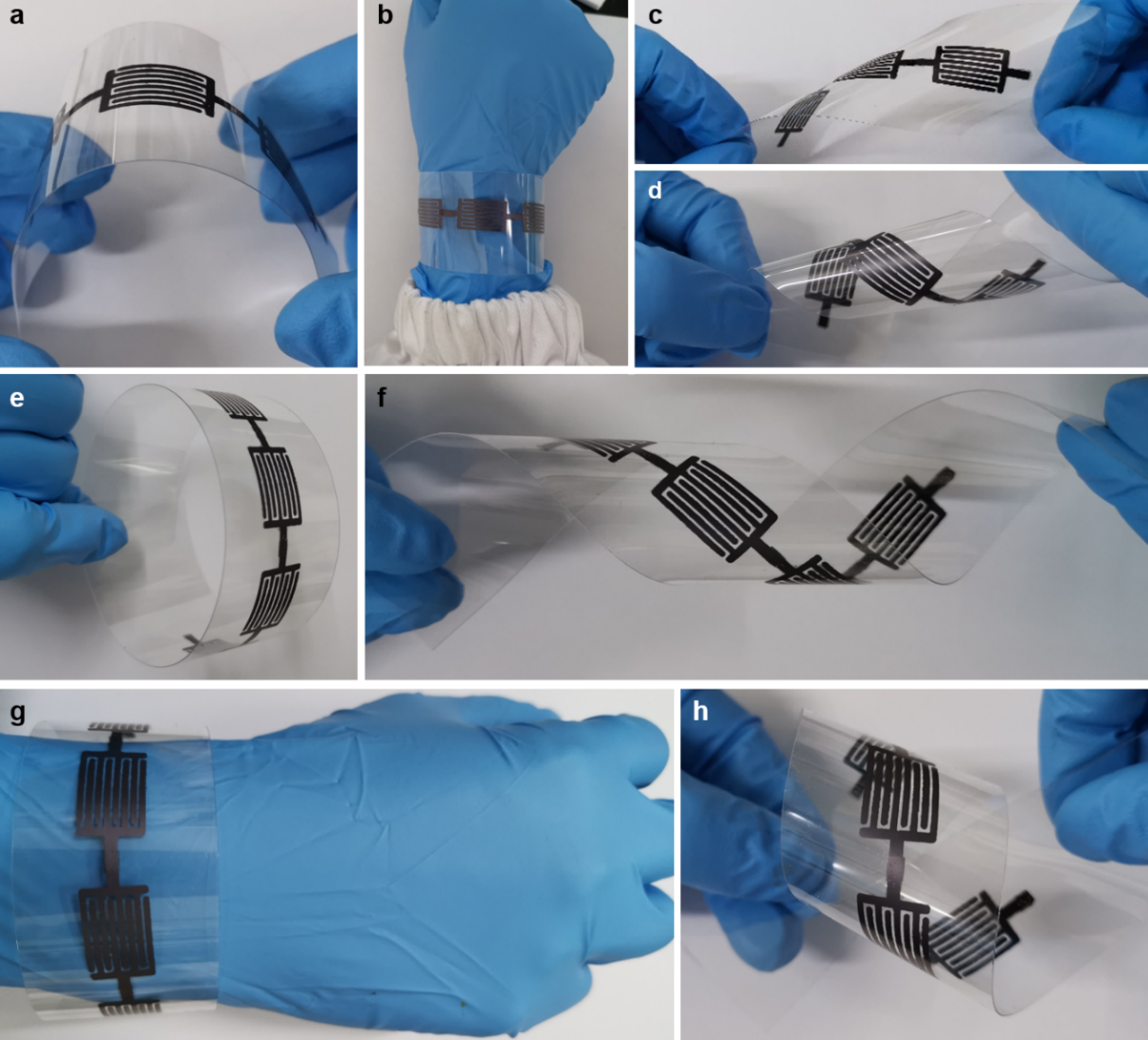
**

**Figure S22.** Integration and flexibility of MXene-MSCs. Photographs of three serially connected MXene-MSCs in (a, b) bending and (c, d) twisting states for the three in series; Photographs of three serially connected MXene-MSCs in (e) bending and (f) twisting states for the four in series.


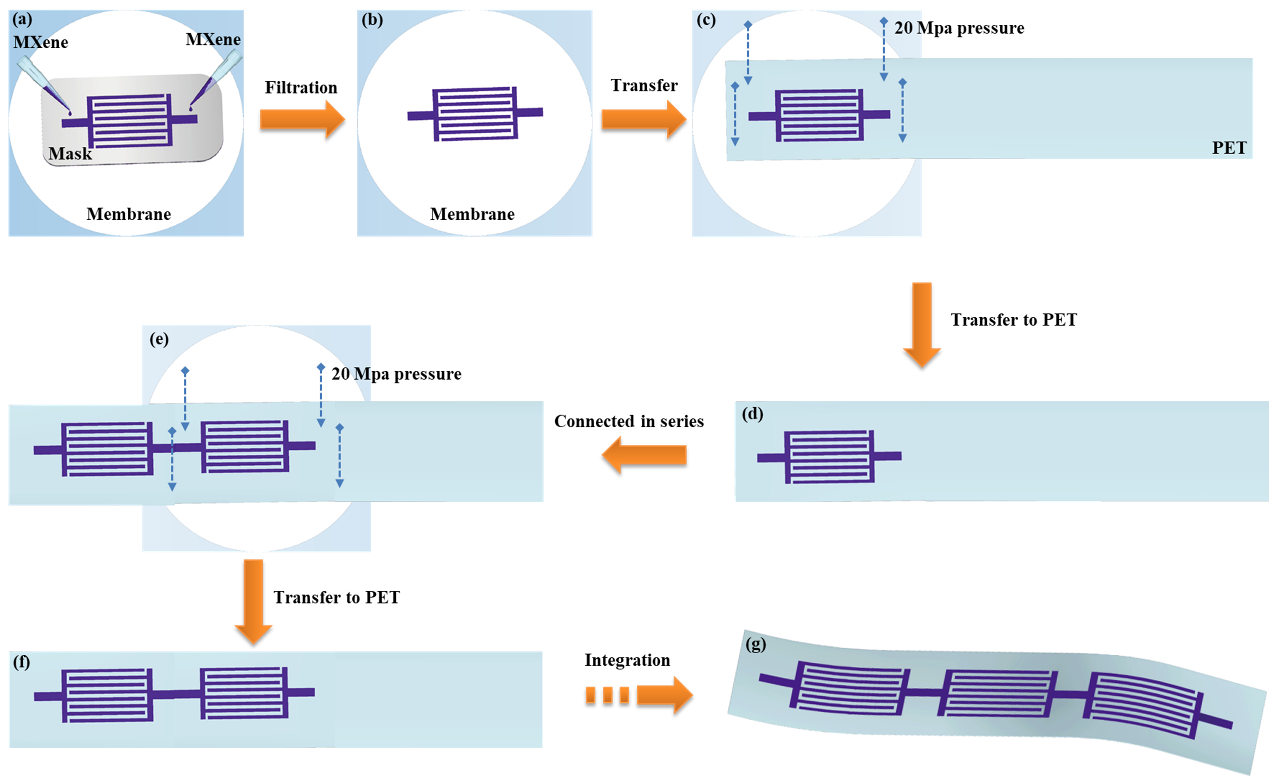


**Figure S23.** The scheme of the fabrication of MXene-MSCs connected in series through a one-by-one transfer strategy. Firstly, MXene dispersion was uniformly filtrated through a mask to form an interdigital MXene microelectrode on the polyvinylidene fluoride (PVDF) membrane (**Figure S23a**). After the removal of the mask, the as-fabricated interdigital microelectrode (**Figure S23b**) was fully transferred on a flexible polyethylene terephthalate (PET) substrate under the pressure of 20 MPa (**Figure S23c**). Then, the PVDF membrane was peeled off to get a single MSC (**Figure S23d**). Second, one end of the filtered MXene microelectrode on the PVDF membrane is overlapped with one end of the other MXene microelectrode on the PET, and transferred to the PET under the same pressure (**Figure S23e**). Afterwards, the PVDF membrane was peeled off to obtain serially-connected two MSCs (**Figure S23f**). The integration of multiple serially-connected MSC packs (**Figure S23g**) could be achieved through this one-by-one transfer strategy. Moreover, the MSC pack could be easily connected in parallel through the same transfer strategy, without any metal-based interconnects and contacts.


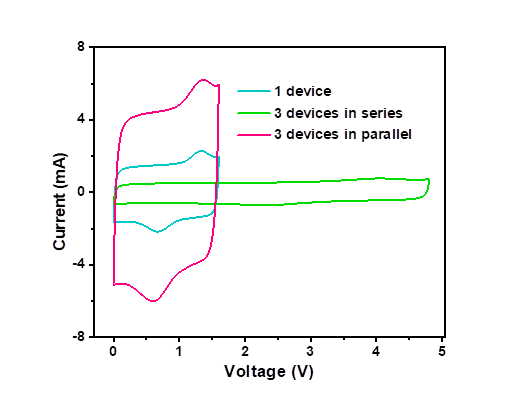


**Figure S24.** Integration and electrochemical performance of MXene-MSCs. CV curves were obtained at 50 mV s^−1^ of single, series, and parallel connections of three MXene-MSCs in 20 m LiCl-gel electrolyte. The series connection enables the extension of the output voltage to 4.8 V (triple the 1.6 V for a single device). Meanwhile, the current can readily multiply by adapting the parallel connection, demonstrating advanced modulation for device integration.

**Table S1.** The prices of salts used in the reported works, obtained from different chemical reagent companies

| **Salts** | **CAS No.** | **Companies** | | | | | | | |
| --- | --- | --- | --- | --- | --- | --- | --- | --- | --- |
|  |  | **Sigma Aldrich** | | | | **Aladdin** | | | |
|  |  | **Stock No.** | **Pack Size** | **Price ($)** | **Price (￥)** | **Stock No.** | **Pack Size** | **Price ($)** | **Price (￥)** |
| LiTFSI | 90076-65-6 | 449504 | 10 g | 125.95 | 874.06 | B102576 | 10 g | 37.32 | 259 |
|  |  |  | 50 g | 430.92 | 2,990.45 |  | 50 g | 73.35 | 509 |
| LiOTf | 33454-82-9 | 282669 | 25 g | 102.10 | 708.54 | L101046 | 25 g | 69.02 | 479.00 |
|  |  |  | 100 g | 359.92 | 2,497.74 |  | 100 g | 216.01 | 1,499.00 |
| LiBETI | 132843-44-8 | - | - | - | - | L157765 | 250 mg | 43.09 | 299 |
|  |  |  | - | - | - |  | 1 g | 83.43 | 579 |
| Pyr_14_·TFSI | 223437-11-4 | 900873 |  | 0.00 |  | B101512 | 5g | 63.26 | 439 |
|  |  |  | 25 g | 1104.95 | 7,667.95 |  | 25g | 230.42 | 1,599.00 |
| Me_3_EtN·TFSI | Laboratory made/ Customization | - | - | - | - | - | - | - | - |
|  |  |  | - | - | - |  | - | - | - |
| EMIMBF_4_ | 143314-16-3 | 00768 | 5 g | 268.17 | 1,861.03 | E120493 | 5 g | 28.68 | 199 |
|  |  |  | 50 g | 747.18 | 5,185.17 |  | 25 g | 77.67 | 539 |
| Et_4_NBF_4_ | 429-06-1 | T113707 | 5 g | 25.79 | 179 | 86618 | 5g | 137.27 | 952.6 |
|  |  |  | 25 g | 77.53 | 538 |  | 25g | 480.35 | 3,333.43 |
| LiCl | 7447-41-8 | 310468 | 100 g | 93.75 | 650.56 | L298760 | 100 g | 14.27 | 99 |
|  |  |  | 500 g | 273.68 | 1,899.21 |  | 500 g | 43.09 | 299 |

**Table S2.** The cost of salt with different concentrations in 1 g electrolyte

| **Salts** | **Molecular Weight**  **(g mol^−1^)** | **Concentration**  **(mol Kg^−1^)** | **Weight**  **(g)** | **Unit Price**  **(USD g^−1^)** | **Cost**  **(USD)** | **Cost**  **(CNY)** |
| --- | --- | --- | --- | --- | --- | --- |
| LiTFSI [2] | 287.09 | 21 | 0.86 | 12.60 | 10.8 | 74.97 |
| 28 m WIBS (21 m LiTFSI + 7 m LiOTf) [3] | 287.09&156.01 | 21&7 | 0.74&0.13 | 12.60&4.08 | 9.9 | 68.72 |
| Li(TFSI)_0.7_(BETI)_0.3_·2H_2_O [4] | 287.09&387.09 | 27.8 | 0.57&0.33 | 12.60&83.43 | 34.6 | 240.2 |
| 63 m WIHS (42 m LiTFSI + 21 m Pyr_14_·TFSI) [5] | 287.09&422.41 | 42&21 | 0.55&0.40 | 12.60&44.20 | 24.8 | 172.2 |
| 63 m WIHS (42 m LiTFSI + 21 m Me_3_EtN·TFSI) [5] | 287.09&368.33 | 42&21 | 0.58&0.37 | 12.60&100 | 44.5 | 308.9 |
| LiCl | 42.39 | 20 | 0.46 | 0.94 | 0.43 | 3.0 |

The prices of the smallest pack size of salts from Sigma Aldrich were chosen to calculate the cost of the electrolytes. The water cost of aqueous electrolytes was ignored in the above calculation. The cost of Me_3_EtN·TFSI in the above calculation was estimated by the synthesis cost of the laboratory.

Specifically, the salt price of 20 m LiCl electrolyte except water is evaluated to be 0.43 $ g^−1^, much cheaper than that of the imide-based WIS electrolytes (Table S2), such as 21 m LiTFSI (10.8 $ g^−1^) [2], 27.8 m Li(TFSI)_0.7_(BETI)_0.3_·2H_2_O (34.6 $ g^−1^) [4], 28 m WIBS (21 m LiTFSI + 7 m LiOTf, 9.9 $ g^−1^) [3], 63 m WIHS (42 m LiTFSI + 21 m Pyr14·TFSI, 24.8 $ g^−1^) [5], and 63 m WIHS (42 m LiTFSI + 21 m Me_3_EtN·TFSI, 44.5 $ g^−1^) [5]. Since a large quantity of solute is required per device, balancing the trade-off between cost and performance is essential.

**Table S3.** Performance comparison of our MXene-MSCs with the state-of-the-art MXene-based MSCs and other symmetric MSCs

| **Device Electrodes** | **Electrolyte** | **Voltage**  **V** | ***C_A_***  **mF cm^-2^** | ***C_V_***  **F cm^-3^** | **E*_A_***  **μWh cm^-2^** | **P*_A_***  **mW cm^-2^** | **E*_V_***  **mWh cm^-3^** | **P*_V_***  **mW cm^-3^** | **Refs.** |
| --- | --- | --- | --- | --- | --- | --- | --- | --- | --- |
| Printing-Ti_3_C_2_T*_x_* | H_2_SO_4_-PVA | 0.5 | 11 | - | 0.3166 | 0.0114 | - | - | [6] |
| EGMX 1:3 | H_3_PO_4_-PVA | 0.8 | 3.26 | 33 | - | - | - | - | [7] |
| 2D Ti_3_C_2_ | H_2_SO_4_-PVA | 0.6 | 6.2 | - | 0.21 | 3.34 | - | - | [8] |
| Clay-like 2D Ti_3_C_2_ | H_2_SO_4_-PVA | 0.6 | 25 | - | 0.77 | 46.6 | - | - | [8] |
| SC-G-40 | H_3_PO_4_-PVA | 1 | - | 17.8 | - | - | 2.47 | 40320 | [9] |
| Printing GO/PA-PE | H_3_PO_4_-PVA | 0.8 | 17.5 | 37.8 | 1.7 | 4.55 | 3.36 | 9820 | [10] |
| Printed δ-MnO_2_ MSC | LiCl-PVA | 0.8 | 0.26 | 2.4 | - | - | 0.18 | 18 | [11] |
| L-s-Ti_3_C_2_T*_x_* MSC | H_2_SO_4_-PVA | 0.6 | 27.3 | 337.5 | - | - | 18 | 15000 | [12] |
| rGO | H_2_SO_4_-PVA | 1 | - | 41.8 | 7.6 | 29.2 | 7 | 30 | [13] |
| UPSCs-25 | H_2_SO_4_-PVA | 1 | 0.87 | 348 | - | - | 12 | 4386000 | [14] |
| SA-MXene | H_2_SO_4_-PVA | 1 | 108.1 | 720.7 | - | - | 100.2 | 1900 | [15] |
| PEDOT-Ti_3_C_2_T*_x_* | H_2_SO_4_-PVA | 0.6 | 2.4 | 240 | - | - | 8.7 | 550 | [16] |
| Ti_3_C_2_T*_x_* | H_2_SO_4_-PVA | 0.6 | 2100 | - | 24.4 | 0.64 | - | - | [17] |
| RuO_2_·*x*H_2_O@MXene-Ag | KOH-PVA | 0.6 | - | 864.2 | - | - | 13.5 | 48500 | [18] |
| Ti_3_C_2_T*_x_*//Ti_3_C_2_T*_x_* | KOH-PVA | 0.6 | 25 | - | 1.25 | 0.11 | - | - | [19] |
| MXene-rGO aerogels | H_2_SO_4_-PVA | 0.6 | 34.6 | - | 2.18 | 0.06 | - | - | [20] |
| Ti_3_C_2_T*_x_* | H_2_SO_4_-PVA | 0.6 | 27.29 | 119 | - | - | 6.10 | - | [21] |
| Ti_3_C_2_T*_x_* MXene | H_3_PO_4_-PVA | 0.6 | 23 | - | 7.8 | - | 2.8 | 225 | [22] |
| Ti_3_C_2_T*_x_* MXene | H_2_SO_4_-PVA | 1 | - | 1.44 | - | - | 0.2 | 144 | [23] |
| Printed Ti_3_C_2_T*_x_* MXene | H_2_SO_4_-PVA | 0.6 | 39.5 | - | 1.64 | 0.7783 | - | - | [24] |
| MXene-MSC 3.2 μm | 20 m LiCl-gel | 1.6 | 28.5 | 89.2 | 10.2 | 0.09 | 31.7 | 250 | **This work** |
| MXene-MSC 8 μm | 20 m LiCl-gel | 1.6 | 71.8 | 89.8 | 25.5 | 0.08 | 31.9 | 100 | **This work** |
| MXene-MSC −40℃ | 20 m LiCl-gel | 1.6 | 20.1 | 62.9 | 7.16 | 0.08 | 22.4 | 250 | **This work** |
| MXene-MSC 60℃ | 20 m LiCl-gel | 1.6 | 30.6 | 95.8 | 10.9 | 0.08 | 34.0 | 250 | **This work** |

**References**

1. Ghidiu M, Lukatskaya MR, Zhao M-Q *et al.* Conductive two-dimensional titanium carbide ‘clay’ with high volumetric capacitance. *Nature* 2014; **516**: 78-81.

2. Suo L, Borodin O, Gao T *et al.* "Water-in-salt" electrolyte enables high-voltage aqueous lithium-ion chemistries. *Science* 2015; **350**: 938-43.

3. Suo L, Borodin O, Sun W *et al.* Advanced high-voltage aqueous lithium-ion battery enabled by “water-in-bisalt” electrolyte. *Angew Chem Int Ed* 2016; **55**: 7136-41.

4. Zheng Q, Miura S, Miyazaki K *et al.* Sodium- and potassium-hydrate melts containing asymmetric imide anions for high-voltage aqueous batteries. *Angew Chem Int Ed* 2019; **58**: 14202-7.

5. Chen L, Zhang J, Li Q *et al.* A 63 m superconcentrated aqueous electrolyte for high-energy Li-ion batteries. *ACS Energy Lett* 2020; **5**: 968-74.

6. Zhang C, McKeon L, Kremer MP *et al.* Additive-free MXene inks and direct printing of micro-supercapacitors. *Nat Commun* 2019; **10**: 1795.

7. Li H, Hou Y, Wang F *et al.* Flexible all-solid-state supercapacitors with high volumetric capacitances boosted by solution processable MXene and electrochemically exfoliated graphene. *Adv Energy Mater* 2017; **7**: 1601847.

8. Kurra N, Ahmed B, Gogotsi Y *et al.* MXene-on-paper coplanar microsupercapacitors. *Adv Energy Mater* 2016; **6**: 1601372.

9. Li L, Secor EB, Chen K-S *et al.* High-performance solid-state supercapacitors and microsupercapacitors derived from printable graphene inks. *Adv Energy Mater* 2016; **6**: 1600909.

10. Liu Y, Zhang B, Xu Q *et al.* Development of graphene oxide/polyaniline inks for high performance flexible microsupercapacitors via extrusion printing. *Adv Funct Mater* 2018; **28**: 1706592.

11. Wang Y, Zhang Y-Z, Dubbink D *et al.* Inkjet printing of δ-MnO_2_ nanosheets for flexible solid-state micro-supercapacitor. *Nano Energy* 2018; **49**: 481-8.

12. Peng Y-Y, Akuzum B, Kurra N *et al.* All-MXene (2D titanium carbide) solid-state microsupercapacitors for on-chip energy storage. *Energy Environ Sci* 2016; **9**: 2847-54.

13. Sun G, An J, Chua CK *et al.* Layer-by-layer printing of laminated graphene-based interdigitated microelectrodes for flexible planar micro-supercapacitors. *Electrochem Commun* 2015; **51**: 33-6.

14. Wu Z-S, Liu Z, Parvez K *et al.* Ultrathin printable graphene supercapacitors with AC line-filtering performance. *Adv Mater* 2015; **27**: 3669-75.

15. Wu C-W, Unnikrishnan B, Chen IWP *et al.* Excellent oxidation resistive MXene aqueous ink for micro-supercapacitor application. *Energy Storage Mater* 2020; **25**: 563-71.

16. Li J, Levitt A, Kurra N *et al.* MXene-conducting polymer electrochromic microsupercapacitors. *Energy Storage Mater* 2019; **20**: 455-61.

17. Yang W, Yang J, Byun JJ *et al.* 3D printing of freestanding MXene architectures for current-collector-free supercapacitors. *Adv Mater* 2019; **31**: e1902725.

18. Li H, Li X, Liang J *et al.* Hydrous RuO_2_-decorated MXene coordinating with silver nanowire inks enabling fully printed micro-supercapacitors with extraordinary volumetric performance. *Adv Energy Mater* 2019; **9**: 1803987.

19. Xu S, Dall’Agnese Y, Wei G *et al.* Screen-printable microscale hybrid device based on MXene and layered double hydroxide electrodes for powering force sensors. *Nano Energy* 2018; **50**: 479-88.

20. Yue Y, Liu N, Ma Y *et al.* Highly self-healable 3D microsupercapacitor with MXene-graphene composite aerogel. *ACS Nano* 2018; **12**: 4224-32.

21. Hu H, Hua T. An easily manipulated protocol for patterning of MXenes on paper for planar micro-supercapacitors. *J Mater Chem A* 2017; **5**: 19639-48.

22. Jiang Q, Wu C, Wang Z *et al.* MXene electrochemical microsupercapacitor integrated with triboelectric nanogenerator as a wearable self-charging power unit. *Nano Energy* 2018; **45**: 266-72.

23. Shen B-S, Wang H, Wu L-J *et al.* All-solid-state flexible microsupercapacitor based on two-dimensional titanium carbide. *Chin Chem Lett* 2016; **27**: 1586-91.

24. Abdolhosseinzadeh S, Schneider R, Verma A *et al.* Turning trash into treasure: additive free MXene sediment inks for screen-printed micro-supercapacitors. *Adv Mater* 2020; **32**: 2000716.
